# Effects of Perceived Stress and Insomnia on Emotional Intelligence of Medical Students

**DOI:** 10.7759/cureus.94715

**Published:** 2025-10-16

**Authors:** Deeksha Jawale, Rita Khadkikar, Mrunal Sawant, Shwetangi Shinde

**Affiliations:** 1 Physiology, Mahatma Gandhi Mission (MGM) Medical College, Navi Mumbai, IND; 2 Community Medicine, Lokmanya Tilak Medical College, Mumbai, IND

**Keywords:** emotional intelligence, emotional regulation, insomnia, medical students, perceived stress

## Abstract

Introduction

Medical students, our future doctors and an important part of the healthcare team, need good emotional regulation. They face high academic and psychosocial stress due to the curriculum, which puts them at risk for insomnia. Stress and insomnia can negatively affect emotional intelligence (EI). Our study aims to understand the correlation between perceived stress (PS), insomnia, and EI.

Materials and methods

After institutional ethics committee approval and obtaining informed consent, 286 medical students in the age group of 18-25 years were included. Those with chronic diseases and on treatment were excluded. Participants filled out a demographic questionnaire and Cohen’s Perceived Stress Scale (PSS), Insomnia Severity Index (ISI), and Mohapel’s Emotional Intelligence Test (EIT) questionnaires. The data was analyzed using R Statistical Software (R Core Team, 2024), and a p-value of <0.05 was considered statistically significant.

Results

Moderate perceived stress was seen in 74.48% of the participants, and insomnia in 55.94%. EI was mainly in the low category for emotional awareness (EA) (66.78%) and emotional management (EM) (55.59%). A statistically significant association was found between perceived stress and insomnia (p < 0.001), perceived stress and EI (emotional management (p < 0.001), social emotional awareness (SEA) (p = 0.006), and relationship management (RM) (p < 0.001)), and insomnia and EI (emotional awareness (p < 0.001), emotional management (p = 0.008), and relationship management (p = 0.034)).

Conclusion

Higher perceived stress and insomnia severity were associated with lower EI in medical students. Educational and psychological interventions are required to support medical students for stress management and better emotional regulation.

## Introduction

A higher emotional intelligence (EI) helps in self-care, building stronger relationships, and achieving career and personal goals, which medical graduates need as part of the healthcare team [1]. Medical students face significant academic and psychosocial stress due to the curriculum, which puts them at risk for insomnia [2]. This can lead to negative consequences on the overall health of these students. Some of them include difficulty in handling routine situations, poor relationships with friends and family, mild to moderate levels of cognitive dysfunction induced by anxiety, and improper sleep [3].

Stress has been found to exert an influence on the quality of sleep, as well as levels of daytime sleepiness [4]. The National Sleep Foundation recommends seven to nine hours of sleep per night for young adults for proper restoration of physical and mental health [5]. Insomnia is the most common sleep disorder and comprises difficulty in falling or staying asleep, despite having sufficient opportunity to sleep, and is associated with daytime dysfunction [6].

For medical students, skill development and productive daily functioning are crucial, which can be influenced by good quantity and quality of sleep. Lack of sleep has also been linked to mood swings, weakened cognitive function, and lessened ability to integrate emotions [7].

Goleman defined EI as the ability to recognize and regulate emotions in oneself and others. This was explained by four domains: self-awareness, the ability to recognize and understand one’s own emotions; self-management, the ability to control one’s emotions and behavior, and adapt to challenging circumstances; social awareness, the ability to sense, understand, and respond to others’ emotions; and relationship management, the ability to inspire, influence, and build relationships with others and help to manage conflicts [1].

Both sleep and stress have been individually associated with trait EI. Higher severity of insomnia is related to a lower score on all four aspects of EI, and an inverse correlation has been reported between perceived stress (PS) and EI [8,9]. Perceived stress is the degree to which life situations are appraised as stressful.

Self-blame, behavioral disengagement, and substance abuse as coping strategies have been reported in students suffering from high PS [10]. Chronic stress has been linked to lifestyle disorders such as diabetes mellitus, hypertension, poor immune response, and cancer [11,12]. High academic stress contributes to psychological distress, which could also have personal and professional ramifications such as medical errors, malpractice lawsuits, and even suicidal thoughts [13]. This study aimed to observe the relationships between perceived stress, insomnia severity, and specific dimensions of emotional intelligence among medical students.

## Materials and methods

This observational, cross-sectional study was conducted in the department of physiology at a medical college in Navi Mumbai, India, following approval from the institutional ethics committee (approval number: DHR-EC/2025/01/13). The study period was from January to May 2025.

The study aimed to assess PS through the Cohen’s Perceived Stress Scale-10 (PSS-10), insomnia severity through the Insomnia Severity Index (ISI), and EI using the Mohapel’s Emotional Intelligence Test (EIT) in medical students and observe their associations with each other [14-16].

Based on previous studies that showed the correlation between PS and EI of about -0.2, our sample size was calculated to be 191. Assuming a 10% non-response rate, our target for the study was 231 participants. The inclusion criteria were first- and second-year medical students in the age group of 18-25 years who consented to the study. Our study invited 350 students using convenience sampling, of which 39 were non-responders and 25 were excluded based on the exclusion criteria (renal or respiratory disorders, diabetes mellitus, any diagnosed mental health conditions, and sleep disorders on treatment). Finally, 286 participants were included in the study.

Participants filled a demographic case study form and the three questionnaires (PSS-10, ISI, and EIT) under supervision, with scoring done and grouped as per the established guidelines.

The demographic record form contained details such as age, gender, year of study, primary stressor (academic or personal), and two self-rated Likert scales scoring quality of sleep and overall levels of stress (from 1 to 5). For quality of sleep, scores less than 3 were categorized as poor, 3 as moderate, and more than 3 as good quality of sleep. Similarly, overall stress levels were classified as low (less than 3), moderate (3), and high (more than 3).

Cohen’s PSS-10 consisted of 10 questions that asked the participants about their thoughts and feelings in the last month, the total score of which ranged from zero to 40 [14]. Our study subjects responded on a 5-point scale ranging from zero (never) to 4 (very often). Out of the 10 questions, four were worded in a positive direction and, hence, were reverse-scored. The scores were categorized as follows: low (<13), moderate (14-26), and high (>26).

ISI is a seven-item scale (rated from zero to 4 each) that includes questions regarding sleep, observed by self and others [15]. The recall period asked in the questions was two weeks. It assessed the nature, intensity, and effect of insomnia. The scores ranged from zero to 28, which was interpreted as no clinically significant insomnia (0-7), subthreshold insomnia (8-14), moderate clinical insomnia (15-21), and severe clinical insomnia (22-28).

Mohapel’s EIT consisted of 10 questions in each of the four components (total: 40 questions) of emotional intelligence: relationship management (RM), social emotional awareness (SEA), emotional awareness (EA), and emotional management (EM), with a score of zero to 4 assigned to each question [16]. The scores for each component were referred to as follows: less than 24 as “low” (area for enrichment), 25-34 as “medium” (effective functioning), and 35-40 as “high” (enhanced skills).

Statistical analysis

Descriptive statistics were performed, and graphs and tables were prepared using Microsoft Excel (Microsoft Corp., Redmond, WA). Frequencies (number) and percentages (%) were calculated for categorical variables, such as age group, sex, year of study, main stressor, perceived stress level, insomnia severity, and emotional intelligence (EI) domains.

Analyses, such as Chi-square and Fisher’s tests, were performed in R Statistical Software (R Core Team, 2024), using a p-value of <0.05 to infer statistical significance [17].

## Results

Among the 286 participants, most were 18-20 years old, with a higher proportion of female students (180) than male students (106). The primary reason for stress among medical students was academic stress, followed by a combination of academic and personal reasons (Table 1).

**Table 1:** Demographic characteristics of the participants

Parameter	Category	Frequency (N = 286)	Percentage (100%)
Age (years)	18	81	43.55%
	19	77	41.40%
	20	19	10.22%
	21	7	3.76%
	22	2	1.08%
Sex	Female	180	62.94%
	Male	106	37.06%
Year of study	First year	169	59.10%
	Second year	117	40.90%
Main stressor	Academic	145	50.70%
	Personal	21	7.34%
	Both	120	41.96%

Overall, on a self-rated scale of 1 to 5 reporting quality of sleep, 40.21% of the students reported poor sleep, while only 20.63% reported having good sleep. Significant differences in sleep quality were observed by age (p = 0.04) and year of study (p = 0.04), but not by sex (p = 0.35). Thus, poor sleep quality is observed among medical students, particularly among the younger-aged and first-year students, potentially due to academic transition stress.

On a self-rated scale of 1 to 5, 44.41% of the respondents stated that they experienced low stress, while 18.18% reported high stress. Stress levels varied significantly by gender (p = 0.007), with male students reporting higher stress. Age and academic year did not show significant associations with overall stress.

The mean and standard deviations of all three scales (PSS-10, ISI, and subscales of EIT) are given in Table 2. PS was moderate in the majority of students (74.48%). Insomnia severity was observed to be none in 44.06%, subclinical in 38.46%, moderate in 15.38%, and severe in 2.10%. EI was majorly in the low category for EA (66.78%) and EM (55.59%). Most students were in the medium range for SEA. Insomnia and moderate stress were prevalent among the medical students, along with low scores in key EI domains.

**Table 2:** Mean and standard deviations of the scales and subscales

Category	Mean	Standard deviation
Perceived Stress Scale	20.51 out of 40	5.71
Insomnia Severity Index	8.96 out of 28	5.82
Emotional awareness	22.21 out of 40	6.02
Social emotional awareness	27.79 out of 40	7.08
Emotional management	23.91 out of 40	7.45
Relationship management	26.03 out of 40	7.93

On Cohen’s PSS-10 scale, PS levels and sex were found to be significantly correlated (p = 0.002). Among our participants, female students were more likely than male students to experience high levels of perceived stress (73.91% of the high-stress group were female). Age and academic year had no significant relationship with PS levels.

On ISI, a statistically significant high risk of severe insomnia was observed in younger-aged students (p = 0.02) and those in the first year of study (p = 0.002), but not between sex and insomnia severity. These results were similar to those seen on the self-reported scale. Analysis revealed that a greater proportion of male students scored higher in the emotional management domain as compared to female students (p = 0.01).

The association between perceived stress and insomnia severity was statistically significant (p < 0.001), indicating that students with higher levels of PS had severe insomnia (Table 3).

**Table 3:** Association between perceived stress and insomnia severity

Perceived stress	Insomnia severity				Total	p-value	Chi-square statistic
	None (number, %)	Mild (number, %)	Moderate (number, %)	Severe (number, %)			
Low	24 (19.05)	2 (1.82)	1 (2.27)	0 (0.00)	27	<0.001	42.446
Moderate	92 (73.02)	88 (80.00)	31 (70.45)	2 (33.33)	213		
High	10 (7.94)	20 (18.18)	12 (27.27)	4 (66.67)	46		
Total	126 (44.05)	110 (38.46)	44 (15.38)	6 (2.10)	286		

Significant associations were observed between high insomnia severity and low levels of EA (p < 0.001), EM (p = 0.008), and RM (p = 0.034), but not with SEA (p = 0.138). Greater insomnia severity was linked to low EI, with domains such as EA and EM showing the lowest values (Table 4).

**Table 4:** Association of emotional intelligence components with insomnia severity

Emotional intelligence		Insomnia severity				Total (number, %)	p-value	Chi-square statistic
		None (number, %)	Mild (number, %)	Moderate (number, %)	Severe (number, %)			
Emotional awareness	Low	74 (58.73)	80 (72.73)	32 (72.73)	5 (83.33)	191 (66.78)	<0.001	42.466
	Medium	50 (39.68)	29 (26.36)	11 (25.00)	1 (16.67)	91 (31.81)		
	High	2 (1.59)	1 (0.91)	1 (2.27)	0 (0.00)	4 (1.39)		
	Total	126 (44.05)	110 (38.46)	44 (15.38)	6 (2.10)	286 (100)		
Emotional management	Low	55 (43.65)	71 (64.55)	28 (63.64)	5 (83.33)	159 (55.59)	0.0082	17.656
	Medium	53 (42.06)	33 (30.00)	15 (34.09)	1 (16.67)	102 (35.66)		
	High	18 (14.29)	6 (5.45)	1 (2.27)	0 (0.00)	25 (8.74)		
	Total	126 (44.05)	110 (38.46)	44 (15.38)	6 (2.10)	286 (100)		
Social emotional awareness	Low	34 (26.98)	42 (38.18)	12 (27.27)	2 (33.33)	90 (31.46)	0.1388	9.6793
	Medium	65 (51.59)	54 (49.09)	28 (63.64)	2 (33.33)	149 (52.09)		
	High	27 (21.43)	14 (12.73)	4 (9.09)	2 (33.33)	47 (16.43)		
	Total	126 (44.05)	110 (38.46)	44 (15.38)	6 (2.10)	286 (100)		
Relationship management	Low	40 (31.75)	54 (49.09)	22 (50.00)	3 (50.00)	119 (41.60)	0.0338	12.824
	Medium	61 (48.41)	46 (41.82)	19 (43.18)	2 (33.33)	128 (44.75)		
	High	25 (19.84)	10 (9.09)	3 (6.82)	1 (16.67)	39 (13.63)		
	Total	126 (44.05)	110 (38.46)	44 (15.38)	6 (2.10)	286 (100)		

Significant negative associations were also found between PS and EM (p < 0.001), SEA (p = 0.006), and RM (p < 0.001), while EA showed a non-significant trend (p = 0.117). Thus, lower EI scores were seen in participants with high stress (Table 5).

**Table 5:** Association of emotional intelligence components with perceived stress levels

Emotional intelligence		Perceived stress			Total (number, %)	p-value	Chi-square statistic
		Low (number, %)	Moderate (number, %)	High (number, %)			
Emotional awareness	Low	13 (48.15)	143 (67.14)	35 (76.09)	191 (66.78)	0.1174	6.8486
	Medium	13 (48.15)	67 (31.46)	11 (23.91)	91 (31.81)		
	High	1 (3.70)	3 (1.41)	0 (0.00)	4 (1.39)		
	Total	27 (9.44)	213 (74.48)	46 (16.08)	286 (100)		
Emotional management	Low	0 (0.00)	119 (55.87)	40 (86.96)	159 (55.59)	<0.001	96.128
	Medium	13 (48.15)	83 (38.97)	6 (13.04)	102 (35.66)		
	High	14 (51.85)	11 (5.16)	0 (0.00)	25 (8.74)		
	Total	27 (9.44)	213 (74.48)	46 (16.08)	286 (100)		
Social emotional awareness	Low	3 (11.11)	79 (37.09)	8 (17.39)	90 (31.46)	0.0059	13.461
	Medium	18 (66.67)	100 (46.95)	31 (67.39)	149 (52.09)		
	High	6 (22.22)	34 (15.96)	7 (15.22)	47 (16.43)		
	Total	27 (9.44)	213 (74.48)	46 (16.08)	286 (100)		
Relationship management	Low	3 (11.11)	89 (41.78)	27 (58.70)	119 (41.60)	<0.001	32.299
	Medium	12 (44.44)	99 (46.48)	17 (36.96)	128 (44.75)		
	High	12 (44.44)	25 (11.74)	2 (4.35)	39 (13.63)		
	Total	27 (9.44)	213 (74.48)	46 (16.08)	286 (100)		

## Discussion

Medical students suffer greater perceived stress when compared with the population at large and students from other academic disciplines [18,19]. The primary reason observed has been academic pressure, which was consistent with our findings [20]. When asked to score “overall stress” on a self-rated scale of 1 to 5, male students mentioned greater stress levels. On Cohen’s PSS-10 scale, however, our results support earlier investigations showing that female medical students experience elevated degrees of PS in comparison to their male peers [21].

A positive correlation between insomnia and stress has been established by prior research, and this study observed that participants with severe insomnia reported higher levels of PS [4,10]. Of our study participants, 40% self-reported as having poor quality of sleep, while on the ISI scale, 55.94% had insomnia, with 17% reporting clinically significant insomnia. This is more than the 10% prevalence of insomnia in the general population, indicating increased sleep difficulties among medical students [22]. A link between insomnia and female sex has been observed in the literature, but no difference was observed in reported sleep quality by gender among our participants [23].

Poor sleepers in medical school have twice the odds of being short sleepers (≤6 hours) during their residency years, with sleep onset insomnia being a strong predictor for anxiety development under stress [24]. This strongly emphasizes the role of sleep in affecting the ability of doctors to handle day-to-day pressures.

The possible cause for an association between insomnia and stress is explained by hyperarousal. This is due to increased cortisol activation caused by psychological stress at bedtime, which is responsible for both acute and chronic insomnia [25].

The analysis also revealed an association between sleep quality and EI. Poor sleepers have lower emotional clarity, management, and overall EI scores [26]. This relationship is bidirectional, as impaired emotional regulation is also related to sleep disturbances [27].

There are previous reported findings of higher PS being linked to lower EI [8]. Those with higher EI are better able to manage their feelings and reactions in response to stressful situations, as they can better regulate their mood, and understand and accurately convey their emotions [28].

Decreased EI is correlated with academic performance in students [29]. This poses a major issue as it leads to a cycle of academic pressures leading to high perceived stress, which is in turn associated with a lower EI, and worse academic outcomes. However, EI is not unchanging. Educational and psychological interventions based on EI could significantly improve students’ emotional management skills and reduce their academic stress [13,30].

Our study used validated psychological scales and appropriate data analysis methods, giving clear associations between subjective domains such as stress and emotional intelligence.

Limitations

This research was based on students reporting their behaviors related to sleep, emotional regulation, and awareness, and perceived stress, which could have a possible recall bias. Factors such as lifestyle habits, personality traits, and social support were not assessed. They could have influenced stress, sleep, and emotional intelligence. The study was also conducted in a single institution, and further multi-institutional data may be needed.

## Conclusions

Stress and insomnia are prevalent among medical students because of the academic demands they endure, particularly the academic transition stress during their first year of medical school. We observed a significant negative association of EI with PS and insomnia. As EI plays a role in multiple aspects of life, such as personal, academic, and professional, its reduction in the presence of stress and insomnia is a cause for concern.

Educational and psychological strategies are required to support medical students in stress management and better emotional regulation. Initiatives targeting both high stress and poor sleep may have the added benefit of increased emotional resilience, especially during training years. Multi-institutional studies are needed with a focus on interventions to see how stress, sleep, and emotional intelligence further interact with each other and whether the interventions have a long-lasting effect.

## Appendices

The questionnaires for demographic information, exclusion criteria, and self-reported sleep and stress scores, Perceived Stress Scale, Insomnia Severity Index, and Mohapel's Emotional Intelligence Test are presented in Table 6, Table 7, Table 8, and Table 9, respectively. 

**Table 6:** Demographic information, exclusion criteria, and self-reported sleep and stress scores

Items	Responses
Age	
Gender	
Year of study	
Quality of sleep in the past 1 year (1 being good sleep and 5 being poor sleep)	
Overall stress levels in the past year (1 being no stress or little stress and 5 being high stress)	
What is the main stressor for you (academic/personal)?	
Diagnosed mental health/lifestyle/renal/sleep/respiratory disorders	

**Table 7:** Perceived Stress Scale-10 0: never, 1: almost never, 2: sometimes, 3: fairly often, 4: very often Permission obtained from Mapi Research Trust

No.	Statement	Score				
1	In the last month, how often have you been upset because of something unexpected?	0	1	2	3	4
2	In the last month, how often have you felt that you were unable to control the important things in your life?	0	1	2	3	4
3	In the last month, how often have you felt “nervous” and “stressed”?	0	1	2	3	4
4	In the last month, how often have you felt confident about your ability to handle your personal problems?	0	1	2	3	4
5	In the last month, how often have you felt that things were going your way?	0	1	2	3	4
6	In the last month, how often have you found that you could not cope with all the things that you had to do?	0	1	2	3	4
7	In the last month, how often have you been able to control irritations in your life?	0	1	2	3	4
8	In the last month, how often have you felt that you were on top of things?	0	1	2	3	4
9	In the last month, how often have you been angered because of things that were outside of your control?	0	1	2	3	4
10	In the last month, how often have you felt difficulties were piling up so high that you could not overcome them?	0	1	2	3	4

**Table 8:** Insomnia Severity Index Permission obtained from Mapi Research Trust

No.	Question (past 2 weeks)	Score 0	Score 1	Score 2	Score 3	Score 4
1	Difficulty falling asleep	None	Mild	Moderate	Severe	Very severe
2	Difficulty staying asleep	None	Mild	Moderate	Severe	Very severe
3	Problems waking up too early	None	Mild	Moderate	Severe	Very severe
4	Satisfaction with current sleep pattern	Very satisfied	Satisfied	Moderately satisfied	Dissatisfied	Very dissatisfied
5	Noticeability of sleep problem to others	Not at all noticeable	A little	Somewhat	Much	Very much
6	Worry/distress about current sleep problem	Not at all worried	A little	Somewhat	Much	Very much
7	Interference with daily functioning (fatigue, mood, work, concentration, memory, etc.)	Not at all interfering	A little	Somewhat	Much	Very much interfering

**Table 9:** Mohapel’s Emotional Intelligence Test Scores 0, 1, 2, 3, and 4 correspond to never, rarely, sometimes, often, and always, respectively.

Domain	Statement	Score				
Emotional awareness	I am able to stand apart from my thoughts and feelings and examine them.	0	1	2	3	4
	My feelings are clear to me at any given moment.	0	1	2	3	4
	Emotions play an important part in my life.	0	1	2	3	4
	My moods impact the people around me.	0	1	2	3	4
	I find it easy to put words to my feelings.	0	1	2	3	4
	My moods are easily affected by external events.	0	1	2	3	4
	I can easily sense when I’m going to be angry.	0	1	2	3	4
	I readily tell others my true feelings.	0	1	2	3	4
	I find it easy to describe my feelings.	0	1	2	3	4
	Even when I’m upset, I’m aware of what’s happening to me.	0	1	2	3	4
Emotional management	I accept responsibility for my reactions.	0	1	2	3	4
	I find it easy to make goals and stick with them.	0	1	2	3	4
	I am an emotionally balanced person.	0	1	2	3	4
	I am a very patient person.	0	1	2	3	4
	I can accept critical comments from others without becoming angry.	0	1	2	3	4
	I maintain my composure, even during stressful times.	0	1	2	3	4
	If an issue does not affect me directly, I don’t let it bother me.	0	1	2	3	4
	I can restrain myself when I feel anger toward someone.	0	1	2	3	4
	I control urges to overindulge in things that could damage my well-being.	0	1	2	3	4
	I direct my energy into creative work or hobbies.	0	1	2	3	4
Social emotional awareness	I consider the impact of my decisions on other people.	0	1	2	3	4
	I can tell easily tell if the people around me are becoming annoyed.	0	1	2	3	4
	When people’s moods change, I sense it.	0	1	2	3	4
	I am able to be supportive when giving bad news to others.	0	1	2	3	4
	I am generally able to understand the way other people feel.	0	1	2	3	4
	My friends can tell me intimate things about themselves.	0	1	2	3	4
	It is hard for me to see other people suffer.	0	1	2	3	4
	I usually know when to speak and when to be silent.	0	1	2	3	4
	I care what happens to other people.	0	1	2	3	4
	When people’s plans change, I understand.	0	1	2	3	4
Relationship management	I am able to show affection.	0	1	2	3	4
	My relationships are safe places for me.	0	1	2	3	4
	I find it easy to share my deep feelings with others.	0	1	2	3	4
	I am good at motivating others.	0	1	2	3	4
	I am a fairly cheerful person.	0	1	2	3	4
	It is easy for me to make friends.	0	1	2	3	4
	People tell me I am sociable and fun.	0	1	2	3	4
	I like helping people.	0	1	2	3	4
	Others can depend on me.	0	1	2	3	4
	I am able to talk someone down if they are very upset.	0	1	2	3	4
